# The FIB-PPH trial: fibrinogen concentrate as initial treatment for postpartum haemorrhage: study protocol for a randomised controlled trial

**DOI:** 10.1186/1745-6215-13-110

**Published:** 2012-07-17

**Authors:** Anne Juul Wikkelsoe, Arash Afshari, Jakob Stensballe, Jens Langhoff-Roos, Charlotte Albrechtsen, Kim Ekelund, Gabriele Hanke, Heidi Fosgrau Sharif, Anja U Mitchell, Jens Svare, Ane Troelstrup, Lars Møller Pedersen, Jeannet Lauenborg, Mette Gøttge Madsen, Birgit Bødker, Ann M Møller

**Affiliations:** 1 Department of Anaesthesiology and Intensive Care Medicine, Copenhagen University Hospital, Herlev Ringvej 75, DK-2730, Herlev, Denmark; 2 Department of Anaesthesia, Mother and Children section, Juliane Marie Centre, Copenhagen University Hospital, Blegdamsvej 9, DK-2100 KBH Ø, Copenhagen, Rigshospitalet, Denmark; 3 Department of Infectious Disease Control and Prevention and Control, Geneva University Hospital, Rue Gabrielle-Perret-Gentil 4, CH-1211, Geneva, Switzerland; 4 Department of Anaesthesiology, Centre of Head and Orthopaedics, and Section for Transfusion Medicine, Capital Region Blood Bank, Copenhagen University Hospital, Rigshospitalet, Blegdamsvej 9, DK-2100 KBH Ø, Copenhagen, Denmark; 5 Department of Obstetrics, Juliane Marie Centre, Copenhagen University Hospital, Rigshospitalet, Blegdamsvej 9, DK-2100 KBH Ø, Copenhagen, Denmark; 6 Department of Obstetrics and Gynaecology, Copenhagen University Hospital, Herlev Ringvej 75, DK-2730, Herlev, Denmark; 7 Department of Anaesthesiology and Intensive Care Medicine, Copenhagen University Hospital, Kettegård allé 30, DK-2650, Hvidovre, Denmark; 8 Department of Obstetrics and Gynaecology, Copenhagen University Hospital, Kettegård allé 30, DK-2650, Hvidovre, Denmark; 9 Department of Anaesthesiology and Intensive Care Medicine, Copenhagen University Hospital, Dyrehavevej 29, DK-3400, Hillerød, Denmark; 10 Department of Obstetrics and Gynaecology, Copenhagen University Hospital, Dyrehavevej 29, DK-3400, Hillerød, Denmark

**Keywords:** Postpartum haemorrhage, Haemostasis, Blood transfusion, Fibrinogen concentrate, Obstetrics, Thrombelastography, Coagulation

## Abstract

**Background:**

Postpartum haemorrhage (PPH) remains a leading cause of maternal mortality worldwide. In Denmark 2% of parturients receive blood transfusion. During the course of bleeding fibrinogen (coagulation factor I) may be depleted and fall to critically low levels, impairing haemostasis and thus worsening the ongoing bleeding. A plasma level of fibrinogen below 2 g/L in the early phase of postpartum haemorrhage is associated with subsequent development of severe haemorrhage. Use of fibrinogen concentrate allows high-dose substitution without the need for blood type crossmatch. So far no publications of randomised controlled trials involving acutely bleeding patients in the obstetrical setting have been published. This trial aims to investigate if early treatment with fibrinogen concentrate reduces the need for blood transfusion in women suffering severe PPH.

**Methods/Design:**

In this randomised placebo-controlled double-blind multicentre trial, parturients with primary PPH are eligible following vaginal delivery in case of: manual removal of placenta (blood loss ≥ 500 ml) or manual exploration of the uterus after the birth of placenta (blood loss ≥ 1000 ml). Caesarean sections are also eligible in case of perioperative blood loss ≥ 1000 ml. The exclusion criteria are known inherited haemostatic deficiencies, prepartum treatment with antithrombotics, pre-pregnancy weight <45 kg or refusal to receive blood transfusion. Following informed consent, patients are randomly allocated to either early treatment with 2 g fibrinogen concentrate or 100 ml isotonic saline (placebo). Haemostatic monitoring with standard laboratory coagulation tests and thromboelastography (TEG, functional fibrinogen and Rapid TEG) is performed during the initial 24 hours.

Primary outcome is the need for blood transfusion. To investigate a 33% reduction in the need for blood transfusion, a total of 245 patients will be included. Four university-affiliated public tertiary care hospitals will include patients during a two-year period. Adverse events including thrombosis are assessed in accordance with International Conference on Harmonisation (ICH) good clinical practice (GCP).

**Discussion:**

A widespread belief in the benefits of early fibrinogen substitution in cases of PPH has led to increased off-label use. The FIB-PPH trial is investigator-initiated and aims to provide an evidence-based platform for the recommendations of the early use of fibrinogen concentrate in PPH.

**Trial registration:**

ClincialTrials.gov NCT01359878.

## Background

Optimal haemostatic capacity during excessive bleeding reduces mortality, blood loss and the need for transfusions [1]. Pregnancy induces changes in coagulation towards a more procoagulant state with mild thrombocytopenia, increased procoagulant factors and diminished fibrinolysis [2], ultimately serving as physiological protection against postpartum bleeding. However, postpartum haemorrhage (PPH) remains a leading cause of maternal mortality and morbidity worldwide [3]. In Europe, 1.75 % of births are complicated by severe PPH (blood loss > 1000 ml) [4] with the risk of death in western countries at 1:100,000 births [5].

### Hypofibrinogenaemia during postpartum haemorrhage

Acquired hypofibrinogenaemia develops early in relation to fluid resuscitation, imbalanced transfusion of blood components and bleeding [6]. This state of impaired haemostasis also develops in relation to PPH [7-9]: a study of 128 women suffering from PPH (blood loss of approximately 1000 ml) investigated by Charbit *et al*. [7] evaluated the impact of coagulation on the course of bleeding. The fibrinogen plasma level was identified as the only early independent predictor of the subsequent development of severe PPH. A fibrinogen level of below 2 g/L implied a positive predictive value of 100% for the development of severe PPH, whereas a level of below 4 g/L implied a negative predictive value of 76%. ‘Severe PPH’ was defined as: ‘decrease of haemoglobin (Hb) > 4 g/dL (>2.5 mmol/L), transfusion of at least four red blood cell (RBC) units, haemostatic intervention (angiographic embolisation, surgical arterial ligation or hysterectomy) or death’.

### Fibrinogen concentrate

Fibrinogen is essential in the development of a strong and functional blood clot. It is produced in the liver and the average plasma level is 2.0 to 4.5 g/L [10]. In relation to pregnancy (especially the third trimester) the level of fibrinogen rises to an average of 5 g/L [11]. Several studies addressing different clinical contexts of bleeding associate a low level of fibrinogen with increased transfusion of blood products and amount of blood loss during operation [8,10,12-18]. No optimal plasma level of fibrinogen has so far been established [10,19-21]. Fibrinogen concentrate is a commercially available drug produced from human plasma [22]. It undergoes viral inactivation and does not require blood match or thawing prior to use. Traditionally, acquired hypofibrinogenaemia is treated with fresh frozen plasma (FFP) or cryoprecipitate (Cryo) containing 2.5 g [10,19] and 15 g per litre [16] respectively. Both blood products require thawing and crossmatch before infusion. Thus, with FFP a volume of 800 ml would be required to substitute 2 g of fibrinogen, corresponding to 133 ml of Cryo. However, the use of FFP is associated with several transfusion-related complications [23] rendering it a suboptimal treatment for early prevention of fibrinogen deficiency. Cryo is a pooled plasma product with one unit of Cryo exposing the recipient to approximately four to six donors [24].

Only four randomised controlled trials (RCT) investigating fibrinogen concentrate have been published so far. One trial investigated the potential of fibrinogen concentrate to reverse a colloid-induced impairment of haemostasis (that is, coagulopathy) in elective surgical patients undergoing radical cystectomy (n = 20) [25]. Two trials explored elective cardiac surgery (n = 20 [26], n = 31 [27]). One trial included severely bleeding elective surgical patients (n = 43) and investigated partial replacement of FFP with fibrinogen concentrate [28]. None of these studies were designed to evaluate a clinical outcome, but indicated improved haemostasis [25,28] and a potential ability to reduce the rate of blood transfusion [25,27]. To our knowledge, no RCT has been published addressing the effect of fibrinogen in acutely bleeding patients or obstetric conditions. However, several publications have described experience from clinical use of fibrinogen concentrate [8,18,29-34] including a total of 144 cases of severe PPH with massive transfusion. In these cases, treatment with fibrinogen concentrate seems to be rapid and efficient in treating hypofibrinogenaemia without any serious adverse events reported in the obstetrical subpopulation. An incidence of 3.48 thrombotic events per 10^5^ treatments, using 4 g of fibrinogen concentrate in different clinical settings was reported in a recent pharmacosurveillance for the period of 1986 to 2008 in 21 countries [35].

### Need for a trial

The FIB-PPH trial will potentially provide evidence-based knowledge on the feasibility of an early use of fibrinogen concentrate in PPH. Though the trial is not powered to show a potential lifesaving effect, an effective treatment reducing the need of red blood cell transfusion could be beneficial. Allogenic blood transfusions are associated with increased mortality and morbidity [36,37]. Besides the potential risk of disease transmission, an immune modulation caused by the transfusion of allogenic blood seems to increase the incidence of infections, cancer relapse, transfusion-related lung injury [19,21] and non-infectious serious hazards of transfusion [23] and is associated with the phenomena of microchimerism [38,39]. Means to reduce allogenic blood transfusions are currently being widely searched for and examined.

Fibrinogen concentrate is increasingly being used in the treatment of PPH. However, this remains off-label use and is based on a low level of evidence. It is of utmost importance to establish sound evidence before a general implementation in the clinical setting. We need to perform high-level RCTs before a more widespread use of fibrinogen concentrate can be recommended.

Following the previously described work by Charbit *et al*. [7] a high-quality trial addressing this matter is needed in order to secure evidence-based treatment and optimal use of economic resources.

### Objective

The aim of this study is to evaluate whether initial treatment with fibrinogen concentrate reduces the need for allogenic blood transfusion in postpartum haemorrhage (PPH). Other potential benefits and harms will be assessed as secondary outcomes.

## Methods and design

### Overview

We are conducting a multicentre parallel placebo-controlled randomised double-blind clinical trial with 1:1 allocation ratio. The trial is investigator initiated and controlled and good clinical practice (GCP) monitored.

### Inclusion criteria

**Figure 1:**
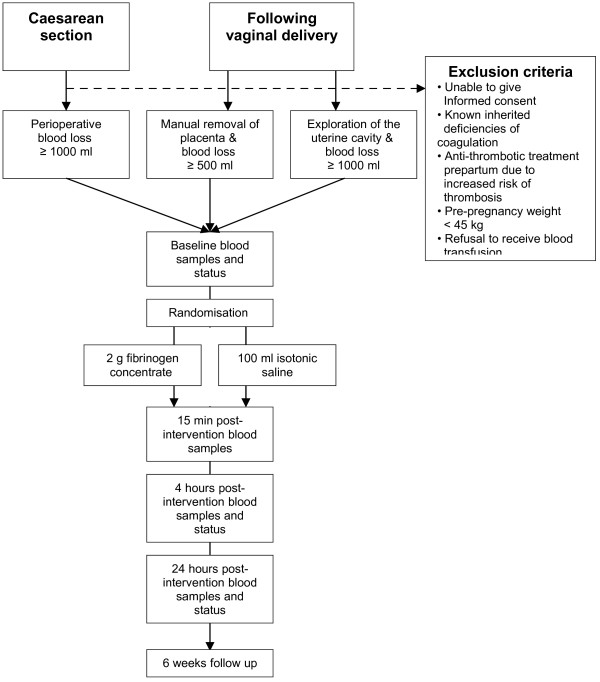
**Trial flow.** Patients scheduled for caesarean section or developing postpartum haemorrhage following vaginal delivery will be screened for inclusion/exclusion and asked for consent to participate. Baseline blood samples are taken before intervention and patients are randomised to either intervention (fibrinogen concentrate) or placebo (saline). Haemostatic monitoring (blood samples) is performed 15 minutes, 4 hours and 24 hours post-intervention together with a clinical status.

Inclusion is possible if the following eligibility criteria are met: 1) Informed consent from participant. 2) Age ≥ 18 years. 3) PPH defined as bleeding from uterus and/or the birth canal within 24 hours postpartum. 4) Vaginal delivery and in need of anaesthesiological assistance and either: a) estimated blood loss exceeding 500 ml and intended manual removal of placenta; or b) estimated blood loss exceeding 1000 ml and intended manual exploration of the uterus due to continuous bleeding after the birth of placenta. 5) In case of birth by caesarean section and estimated perioperative blood loss exceeding 1000 ml (Figure 1).

### Exclusion criteria

Participants fulfilling one or more of the following criteria will be excluded: 1) Known inherited deficiencies of coagulation. 2) Anti-thrombotic treatment prepartum due to increased risk of thrombosis. 3) Pre-pregnancy weight <45 kg. 4) Refusal to receive blood transfusion.

### Randomisation and blinding

Allocation sequence is produced by a third party (pharmacologists at Glostrup Pharmacy, Glostrup, Denmark) using computer generation fixed block size with stratification by centre. Allocation concealment is secured by using sealed opaque numbered envelopes specially prepared for this trial and this unique number is used as patient trial ID. Participants, outcome assessors, trials investigators, statisticians and GCP trial monitors are blinded, as well as care providers such as midwives, obstetricians, anaesthesiologists and anaesthetist nurses. Anaesthetic personnel not involved in patient care randomise the patient and dispense the trial medicine. Two 50 ml opaque syringes (yellow coloured) will be delivered to the operating theatre without revealing the allocation. We register the success of blinding by asking the responsible team/physician in the operating theatre following administration if he or she has any hint of the allocated treatment and if yes, why. The patient is asked during the follow-up period.

### Intervention

The participants are assigned to either 1) placebo (100 ml isotonic saline) i.v. or 2) the intervention drug: 2 g of fibrinogen concentrate (Haemocomplettan/Riastap, CSL Behring, Marburg, Germany) i.v. given via an infusion pump in 20 minutes. We use a fixed dose for all patients randomised to the intervention group without prior clinical assessment of the fibrinogen level. This strategy is primarily based on the clinical urgency since the treatment is required to be administered as early as possible following the inclusion. Dosage is based on a target level of 4 g/L [7] and a mean fibrinogen level in women with 500 to 1000 ml blood loss of 3.4 g/L [9]. Thus, we calculated the optimal dose using two different methods [10,25] in order to increase the level approximately 0.6 g/L in the average 65.9 kg fertile woman in the Danish capital area (based on data from the Danish National Board of Health) taking either the increased plasma volume or weight gain during pregnancy into account. An optimal dose of 2.2 to 2.7 g was identified. However, considering the prothrombotic state of pregnancy and postpartum period and the fact that previous controlled clinical studies investigated patients that were highly diluted with colloids [25] or affected by the coagulopathies of extracorporeal circulation [26,27], we pragmatically chose 2 g of fibrinogen concentrate as the intervention dose.

### Setting, location and follow-up

Parturients will be included during a two-year period at the four largest hospitals in the Capital Region, Denmark. These four centres are university-affiliated public tertiary care hospitals in the Copenhagen area each with 3500 to 7000 deliveries per year. Parturients are invited to participate at the pre-anaesthetic evaluation and will be hospitalised for at least 24 hours following inclusion. Participants are contacted by phone six weeks after the intervention, and they are able to contact the principal investigator during the full trial period. Blood transfusion and the use of tranexamic acid and other haemostatic agents follow Danish standard recommendations in all four centres and adherence to guidelines will be monitored as part of the conduction of this trial.

### Outcomes and safety measures

The primary outcome is the need for transfusion with allogenic blood products. Secondary outcomes will be development of ‘severe PPH’ defined as: ‘decrease of haemoglobin (Hb) of > 4 g/dl (2.5 mmol/L), transfusion of at least four red blood cell (RBC) units, haemostatic intervention (angiographic embolization, surgical arterial ligation or hysterectomy) or death. We also evaluate estimated blood loss, total amount of blood transfused, the development of re-bleeding and Hb < 5.8 g/dL (3.6 mmol/L)’. We will register potential side effects such as: fever, headache, nausea, vomiting, allergic reactions, anaphylaxis and thromboembolic complications (deep venous thrombosis, acute myocardial infarction and pulmonary embolism) [40]. All suspected unexpected serious adverse reactions will also be reported in accordance with the International Conference on Harmonisation (ICH) GCP and the Danish Medicines Agency guidelines.

### Monitoring and data collection

Adherence to ICH GCP, including the Helsinki Declaration, and trial protocol is being monitored by independent monitors from the GCP unit at Copenhagen University, and data are being collected on paper case report forms. Transfusion data are assessed through multiple data sources including the Capital Region Blood Banks blood product registration system in which all used blood products are registered together with the recipient patients’ unique personal identification (cpr) number. This ensures an almost complete follow-up excluding only patients withdrawing consent to participate.

### Haemostatic monitoring

Blood samples are drawn at inclusion before intervention and at 15 minutes, 4 hours and 24 hours following the termination of infusion. Blood transfusions, fluid therapy, use of uterotonic and haemostatic drugs, and amount of blood loss are assessed by anaesthetic personnel before administration, 4 hours and 24 hours after. We use haemostatic tests thrombelastography (TEG, functional fibrinogen and Rapid TEG by Haemoscope Inc., Niles IL, USA) as well as standard tests such as haemoglobin, factor level 2,7,10 (including international normalised ratio (INR)), activated partial thromboplastin time (APTT), D-dimer, antithrombin III and platelet count. Plasma fibrinogen level is measured at all four time points but only the post-interventional measurements will be available for clinical evaluation and treatment aspects. Samples are collected and frozen for later analysis.

### Number of participants

Approximately 1% of parturients receive blood transfusion [7,9,32,41-44] and 1.75% develop PPH (blood loss >1000 ml) [4]. No studies conducted in the obstetric target population were available during the design phase of this trial, however, the two available RCTs found a respectively 60% [25] and 20% but insignificant [26] risk reduction in the need for allogenic blood transfusions. Thus, we settled to investigate a risk reduction of 33%: with α = 0.05 and power (β) = 80%, a number of 107 patients in each group are needed in order to evaluate a 33% reduction in the need for blood transfusion. With a margin for missing data and dropouts of 15% - a total of 245 patients will be included.

### Statistical methods and data analysis

The two groups will be compared using chi-square test (binominal data) or Fischer’s exact test if numbers of expected values are less than five (or an unconditional exact test). Fibrinogen concentrate can potentially reverse the impaired haemostasis observed when using colloids [25,45]. The potential effect of fibrinogen concentrate may rely on the pre-administered use of colloids, so sensitivity analysis and the influence of this will be assessed together with other possible confounders (baseline variables/parameters are presented in Table 1). In accordance with ICH GCP recommendations on statistical principles for clinical trials (E9) [46], we will present adjusted and unadjusted analyses of the possible effect. The baseline variables/parameters will be assessed using multiple logistic regression models. The evaluation of pre-specified subgroups demands more participants than planned in this trial, however, to explore and guide further trials in this area we will perform two subgroups analysis: one on mode of delivery (vaginal versus caesarean section) and one on parturients with hypofibrinogenaemia at intervention, thus guiding upcoming trials in their selection of study population. All outcomes will be reported as ‘intention-to-treat’ analysis including all randomised patients fulfilling inclusion criteria and not meeting exclusion criteria with a standing consent to participate. Patients with the following major protocol violations will not be included in a supplemental per-protocol analysis: not receiving total dose and lack of follow-up. The impact of blinding level will be explored in a separate sensitivity analysis. Statistics will be performed before the code of allocation is revealed. If the overall level of missing data is less than 5%, we will perform complete case analysis. If more than 5% of missing data is the case, we will present ‘worst-case’ and ‘best-case’ analyses and if those are inconclusive, we will perform multiple imputations [47].

**Table 1:** List of baseline parameters/variables to be assessed for baseline imbalance between treatment groups in the final analysis

**Baseline parameter/variable**	
The level of fibrinogen before intervention defined by Clauss method (g/L)	
Presence of hypofibrinogenaemia before intervention defined by Clauss method (<2 g/L) and equivalent functional fibrinogen (TEG) MA (< 14 mm or according to validation in this study).	
Hypocoagulability prior to intervention measured with standard TEG: R-time >8 min or alpha angle <55° or MA <50 mm or LY30 >8 %	
Crystalloids used prior to intervention (ml)	
Colloids used prior to intervention (ml)	
The use of tranexamic acid prior to intervention (yes/no)	
Mode of delivery	Caesarean section
	Vaginal delivery
Post vaginal procedure	Intended manual removal of placenta
	Manual exploration of the uterus due to continuous bleeding after the birth of placenta
Estimated blood loss before intervention (ml)*	
Primary cause of PPH:	
**TRAUMA of the birth canal:** paravaginal haematoma, cervical laceration, vaginal laceration, perineal laceration, uterine rupture	
**Retained TISSUE:** Retained placental tissue, Percrete or accrete placenta	
**Impaired THROMBOSIS:** disseminated intravascular coagulation, placental abruption, severe pre-eclampsia	
**Reduced TONE of uterus:** uterine atony	
Weight of parturient (kg) (because dose of fibrinogen = 2 g/BW kg)	
Units of allogenic blood transfusion transfused prior to intervention (numbers of units)	
Bleeding duration (from start of bleeding to intervention) (min.)	
Time from birth to intervention (min.)	
Baseline haemoglobin (measured before intervention) (mmol/L or g/dL)	
Method of anaesthesia: general versus regional anaesthesia	

Adjusted analysis will be provided and results may guide further research in this field.

### Consent and ethical considerations

Patients will provide informed consent during the pre-anaesthetic assessment. An information brochure is given beforehand to all pregnant women in the Capital Region by midwives at a visit when 29 weeks pregnant, and again to women requiring labour epidural. Before the caesarean section or the post vaginal procedures patients are asked to give their informed consent. Danish law does not provide the option of including incapacitated patients using personal or professional representatives in drug trials if some of the eligible patients are able to give informed consent. The trial is approved by the National Committee on Biomedical Research Ethics 1002168/H-3-2010-004, the Danish Medicines Agency (2612–4233/EudraCT 2009-017736-41) and the Danish Data Protection Agency (2007-58-0015-00911). The trial is registered at clincialtrials.gov (NCT01359878).

## Discussion

This is the first randomised clinical trial investigating the use of fibrinogen concentrate in acutely bleeding patients in an obstetric setting. Furthermore, no previous report has evaluated the initial use of fibrinogen concentrate during acute bleeding and its possible potential effect to reduce the need for blood transfusion. In this trial, we chose to give the same pre-emptive dose of fibrinogen to all participant allocated to the intervention group regardless of body weight and plasma level of fibrinogen in order to secure treatment being given as early as possible. This implies the risk of giving a very low dose to women with high body weight and thereby potentially risking no treatment effect. On the other hand, PPH may be caused by many other factors than impaired haemostasis, such as atony or trauma of the uterus or retained tissue [48]. Once these causes have been treated bleeding usually stops. Thus by giving the same dose to all without prior measurements of the fibrinogen level, we risk giving excess fibrinogen to some. In this trial, close haemostatic monitoring with standard laboratory tests and thrombelastography including functional fibrinogen assay will reveal the haemostatic starting point and monitor any hypercoagulability that might occur.

Up to 1.9% of women giving birth in Denmark receive at least one unit of RBC postpartum [49]. Acquired hypofibrinogenaemia is a well-established cause of bleeding due to impaired haemostasis, but in most countries fibrinogen concentrate is currently approved for inherited hypofibrinogenaemia only. However, a general perception of benefits and potentials of early pre-emptive fibrinogen substitution has led to increased off-label use [34].

The substitution of fibrinogen in patients with acquired hypofibrinogenaemia is well-established as is the essential role of fibrinogen in the achievement of haemostasis in bleeding patients. However, the source of fibrinogen (FFP, Cryo versus fibrinogen concentrate) is still being debated together with the possible benefits and risks of pre-emptive fibrinogen substitution [10,24,50-54]: increasing the fibrinogen level to above normal levels in obstetric patients with bleeding might backfire, since these patients all ready have an increased risk of thrombosis post-bleeding or post-surgery [54]. Several recent reviews address the topic, but the evidence remains poor and inconclusive [10,51-53].

The FIB-PPH trial includes patients with postpartum haemorrhage. Mortality is the ultimate outcome summarising benefits and harms. However, a trial investigating this outcome in an obstetric setting would require an extremely large sample size. An ongoing randomised trial aiming at 15,000 participants worldwide investigates the use of tranexamic acid in postpartum haemorrhage with peripartum hysterectomy as outcome [55]. Trials of this scale require good organisation and funding. Therefore, with the sample size of 245 the FIB-PPH trial should be considered a pilot study, but has the benefit of being an independent investigator-initiated trial.

PPH is a potentially life-threatening acute condition and, therefore, usually not suitable for trial inclusion according to Danish laws and regulations. This could lead to selection bias, as it may be difficult to include the seriously affected women who may very well be the subgroup benefiting most from this intervention. By educating personnel and distributing information material early in pregnancy, we hope to enable the inclusion of the relevant population. Investigation of drugs considered for the treatment of acutely ill patients, such as those with acute bleeding remains a challenge due to the need for maintaining a balance between the patients’ rights for information and consent while at the same time trying to provide evidence-based treatment strategies. The use of personal or professional representatives giving the initial consent is considered legal and practised with success in some countries [55].

A positive result, indicating a potential transfusion sparring effect after births complicated by PPH, could have great importance for developing countries. Fibrinogen concentrate can be stored for five years at room temperature and no thawing or crossmatch is needed. A negative result may indicate that early treatment with a standard dose is potentially insufficient and further research would be needed into thrombelastography-guided treatment algorithms with a tight haemostasis control.

## Trial Status

The trial was initiated in June 2011 with a gradual start at each centre. By November 2011, all four centres were including participants. By June 25, 102 parturients had been included.

We hope to increase the inclusion rate as well as the proportion of patients with severe bleeding as the trial will be fully implemented as part of the daily routine at each hospital in the coming period.

## Abbreviations

APTT, activated partial thromboplastin time; FIB-PPH, fibrinogen as initial treatment of postpartum haemorrhage trial; FFP, fresh frozen plasma; GCP, good clinical practice; Hb, haemoglobin; ICH, International Conference on Harmonisation; INR, international normalised ratio; n, numbers of patients in trial; PPH, postpartum haemorrhage; RBC, red blood cells; RCT, randomised controlled trial; TEG, thrombelastography.

## Competing interests

The authors declare that they have no competing interests. Thrombelastography assays are unrestrictedly sponsored by Haemonetics Inc. Funded by independent grants (see below).

## Author’s contribution

AW, AA, JS, JL-R and AM developed the concept and trial design and secured funding. CA, KE, GH, HFS, AM, JS, AT, LMP, JL, MGM and BB made substantial contributions to the practical issues, logistics and concept of the trial. All were involved in drafting the protocol and revising it critically. All have approved the final version.
